# Genetic association between *FADS* and *ELOVL* polymorphisms and the circulating levels of EPA/DHA in humans: a scoping review

**DOI:** 10.1186/s12263-024-00747-4

**Published:** 2024-06-06

**Authors:** Insaf Loukil, David M. Mutch, Mélanie Plourde

**Affiliations:** 1 Research Center on Aging, Health, and Social Sciences Center, Department of Medicine, Sherbrooke University Geriatrics Institute, University of Sherbrooke, Sherbrooke, QC J1G 1B1 Canada; 2 Department de Medicine, Faculty of Medicine and health sciences, University of Sherbrooke, Sherbrooke, QC J1H 5N4 Canada; 3 Department of Human Health and Nutritional Sciences, Guelph, ON N1G 2W1 Canada

**Keywords:** DHA, *ELOVL*, EPA, *FADS*, SNP

## Abstract

**Background:**

Docosahexaenoic acid (DHA) and eicosapentaenoic acid (EPA) are two omega-3 fatty acids that can be synthesized out of their precursor alpha-linolenic acid (ALA). *FADS* and *ELOVL* genes encode the desaturase and elongase enzymes required for EPA and DHA synthesis from ALA; however, single nucleotide polymorphisms (SNPs) in *FADS* and *ELOVL* genes could modify the levels of EPA and DHA synthesized from ALA although there is no consensus in this area. This review aims to investigate EPA and DHA circulating levels in human blood and their association with *FADS or ELOVL.*

**Methods:**

PubMed, Cochrane, and Scopus databases were used to identify research articles. They were subsequently reviewed by two independent investigators.

**Results:**

Initially, 353 papers were identified. After removing duplicates and articles not meeting inclusion criteria, 98 full text papers were screened. Finally, this review included 40 studies investigating *FADS* and/or *ELOVL* polymorphisms. A total of 47 different SNPs in *FADS* genes were reported. *FADS1* rs174537, rs174547, rs174556 and rs174561 were the most studied SNPs, with minor allele carriers having lower levels of EPA and DHA. SNPs in the *FADS* genes were in high linkage disequilibrium. SNPs in *FADS* were correlated with levels of EPA and DHA. No conclusion could be drawn with the *ELOVL* polymorphisms since the number of studies was too low.

**Conclusion:**

Specific SNPs in *FADS* gene, such as rs174537, have strong associations with circulating levels of EPA and DHA. Continued investigation regarding the impact of genetic variants related to EPA and DHA synthesis is warranted.

## Introduction

**Fig. 1:**
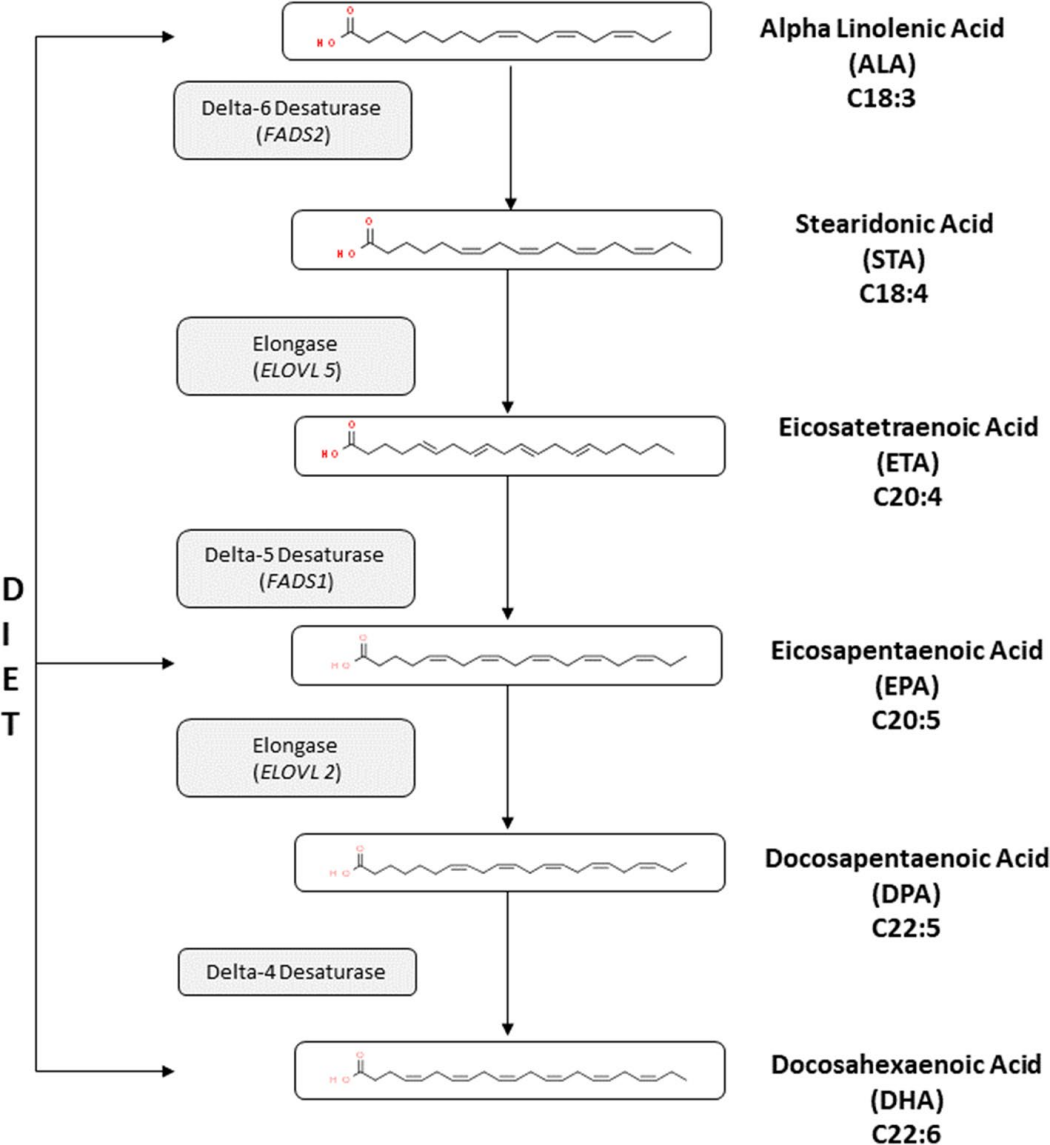
Metabolic pathway of EPA and DHA biosynthesis from ALA via desaturation and elongation steps

Omega-3 fatty acids (ω3) are defined as long chain polyunsaturated fatty acids (LC-PUFA) with 18 or more carbons in length and three or more double bonds [1], where the first double bound is on the third carbon from the methyl end of the acyl chain [2]. Eicosapentaenoic acid (EPA) and docosahexaenoic acid (DHA) are the two main ω3 LC-PUFA studied in the prevention and treatment of a wide range of diseases [3–6]. Higher plasma EPA and DHA levels are usually associated with lower risks of cognitive decline [7, 8] and cardiovascular diseases [9, 10]. EPA and DHA plasma levels are modified by dietary consumption of marine foods, endogenous production of these fatty acids from their essential precursor fatty acid, alpha-linolenic acid (ALA). The synthesis of EPA and DHA from ALA requires sequential enzymatic steps depicted in Fig. 1, and involves desaturation by enzymes encoded by fatty acid desaturase 1 (*FADS1*) and 2 (*FADS2*) genes and elongation via enzymes called elongases encoded by very long chain fatty acids 2 (*ELOVL2)* and 5 (*ELOVL5*) genes [11]. Elongases are encoded by the *ELOVL* gene family located on chromosome 6 [12] (Fig. 1). To date, seven different *ELOVL* genes have been identified in humans [13, 14]. *ELOVL5* is identified as being involved in elongating fatty acids of C18 --» C20 carbons, whereas *ELOVL2* elongates C20 --» C24 fatty acids (Fig. 1) [15]. Desaturation steps are mediated by the enzymes delta-5-desaturase (D5D) and delta-6-desaturase (D6D), which are encoded by *FADS1* and *FADS2* genes, respectively. The *FADS1* and *FADS2* genes are located on human chromosome 11, in a head-to-head arrangement (q12 and q13.1), interspersed by an 11kb region. Another fatty acid desaturase gene, known as *FADS3*, is also located in this *FADS* gene cluster; however, its translated product has not yet been characterized [16]. D6D catalyzes the conversion of ALA into stearidonic acid. An elongation step follows, after which D5D adds a double bond to a 20-carbon fatty acid chain. Eicosatetraenoic acid is then transformed into EPA, via the action of D5D.

Some of the coauthors previously investigated the conversion rate in humans in studies using uniformly labeled carbon 13-ALA, where it was estimated that the rate of conversion of ALA into EPA was ~5% and into DHA was ~0.5 % [17]. The conversion rates can, however, differ depending on sex, age, and menopausal status [18–21]. Dietary intake of ALA, EPA and DHA can also modify the plasma levels of EPA and DHA. In one study, pregnant women who reported consuming foods high in EPA and DHA had higher concentrations of these fatty acids in their blood [22]. In a randomised controlled trial on men and women consuming controlled intakes of 0.25, 0.5, and 1 g/d, blood levels of EPA and DHA increased linearly in whole blood, erythrocytes, and plasma phospholipids with increasing intakes of EPA and DHA [23]. Linoleic acid (LA) can also influence the biosynthesis of EPA and DHA from ALA by competing with ALA as a substrate for the same enzymes, *FADS* and *ELOVL* [24, 25]. One study showed that a low-LA diet increased the conversion of ALA into EPA and DHA by upregulating the expression of *FADS* and *ELOVL* through regulating the expression of transcription factors such as peroxisome proliferator-activated receptor-alpha (PPAR-α) and sterol regulatory element binding protein 1 (SREBP1) [24].

There are other non-modifiable factors that can contribute to lower conversion rate, such as single nucleotide polymorphisms (SNPs) in *FADS* and *ELOVL* genes. *FADS* genes are highly polymorphic with 35 490 single nucleotide polymorphisms (SNPs) located in this gene cluster, as described in the SNP database by the National Center for Biotechnology Information (NCBI) [26]. The search was conducted for each gene individually, and the cumulative sum of single nucleotide variants for *FADS1*, *FADS2*, and *FADS3* was calculated. Among these SNPs, there were 1306 missense and nonsense variants (stop gained)-resulting in a change in the translation of proteins [27].

Previous reviews on SNPs in *FADS* and *ELOVL* focussed on investigating the relationships between these genes and disease risk factors/disease prevalence or were specifically interested in the levels of cholesterol in different lipoproteins (HDL, LDL, VLDL, etc.) [28–31]. The objective of this scoping review was to examine studies that investigated EPA and DHA blood levels in relation to *FADS* and *ELOVL* polymorphisms to estimate their potential contribution to variations in the levels of ω3 LC-PUFA in the bloodstream.

## Methods

A search for studies was conducted in PubMed, Cochrane, and Scopus between March 7th, 2022, and December 21st, 2022 using the following database-specific indexed keywords: "docosahexaenoic acid", "eicosapentaenoic acid", "fatty acids, omega 3", "polymorphism, single nucleotide", "polymorphism, genetic", "SNPs" , "fatty acids, omega 3/metabolism", "fatty acids, omega 3/biosynthesis", "fatty acid desaturases", "fatty acid elongases", "*ELOVL*", "*FADS*" , "Elongase, Fatty Acid" ,"Desaturase, Fatty Acid" , "single nucleotide polymorphism" , "SNP", Eicosapentaenoate" , "Docosahexaenoate" , "omega3" , "polyunsaturated fatty acids" , "omega 3 PUFA" , and "omega 3 fatty acids".

For this review paper, only studies performed in humans were considered. All articles and abstracts were carefully screened by IL. Inclusion criteria were as follows: articles written in English, availability of genetic information for *FADS* and/or *ELOVL* genes, availability of EPA and DHA data either in total plasma, red blood cells (RBC), serum or in different blood compartments such as plasma phospholipids. Studies conducted on maternal milk, umbilical cord venous plasma, or only reporting the calculation of desaturase and elongase indices using product-to-precursor ratios were excluded. Additionally, studies including a supplement or a specific diet in the study were only considered if they presented baseline levels of ω3 LC-PUFA in their data, otherwise they were excluded. Afterwards, eligibility of the selected studies was assessed by IL and MP. Moreover, additional papers were identified from the reference list of relevant articles.

## Results

**Fig. 2:**
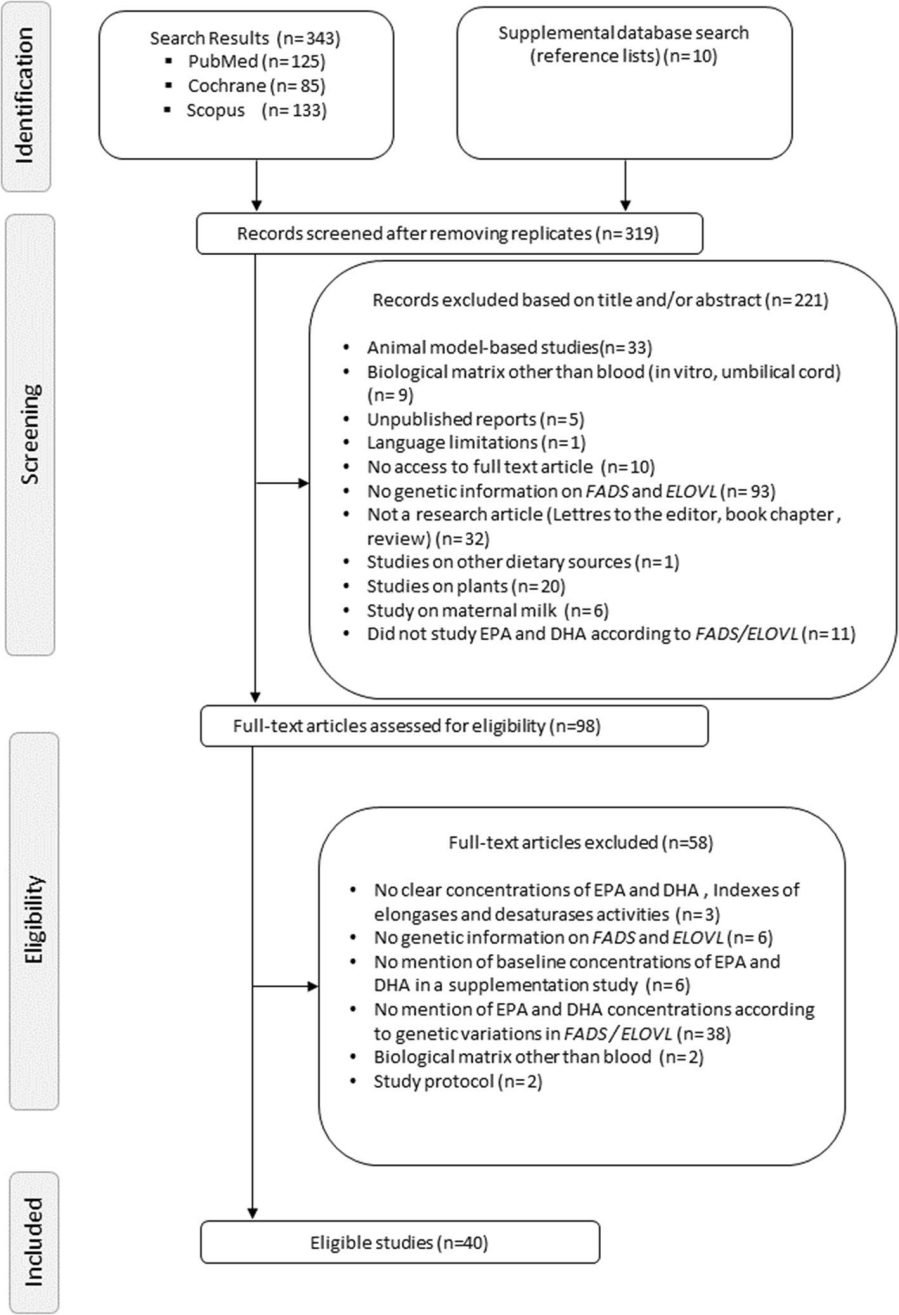
Literature review flowchart

A total of 353 studies were found in the initial search. Duplicates were eliminated from the list, and a further 221 studies were removed because they did not meet inclusion criteria. A total of 98 full-text papers was assessed from which 40 articles were selected for this review (Fig. 2).

The publication dates of the selected studies ranged from 2006 to 2021, with most studies published after 2017 (*n* = 17 studies). Studies were conducted in different age groups including infants, adolescents, adults, and older adults together with pregnant women and post-menopausal women. In addition, participants had different medical histories, ranging from no health issues to participants with heart diseases, type 2 diabetes (T2D), cognitive impairments, psychiatric disorders, obesity, or metabolic syndrome. The selected studies were conducted in various countries around the world: Australia, Brazil, Canada, China, Costa Rica, Denmark, England, France, Germany, Italy, Japan, Mexico, Mahe-island, Netherlands, Poland, Czechia, and South Korea,

The number of participants in the different studies ranged from 12 [32] to 4457 [33], and the mean age ranged from 9 months old [34] to 85 years [35]. There were 22 studies reporting an association between SNPs in *FADS /ELOVL* and EPA/DHA levels.

### *FADS* genetic polymorphisms and EPA/DHA status in blood pools

**Fig. 3:**
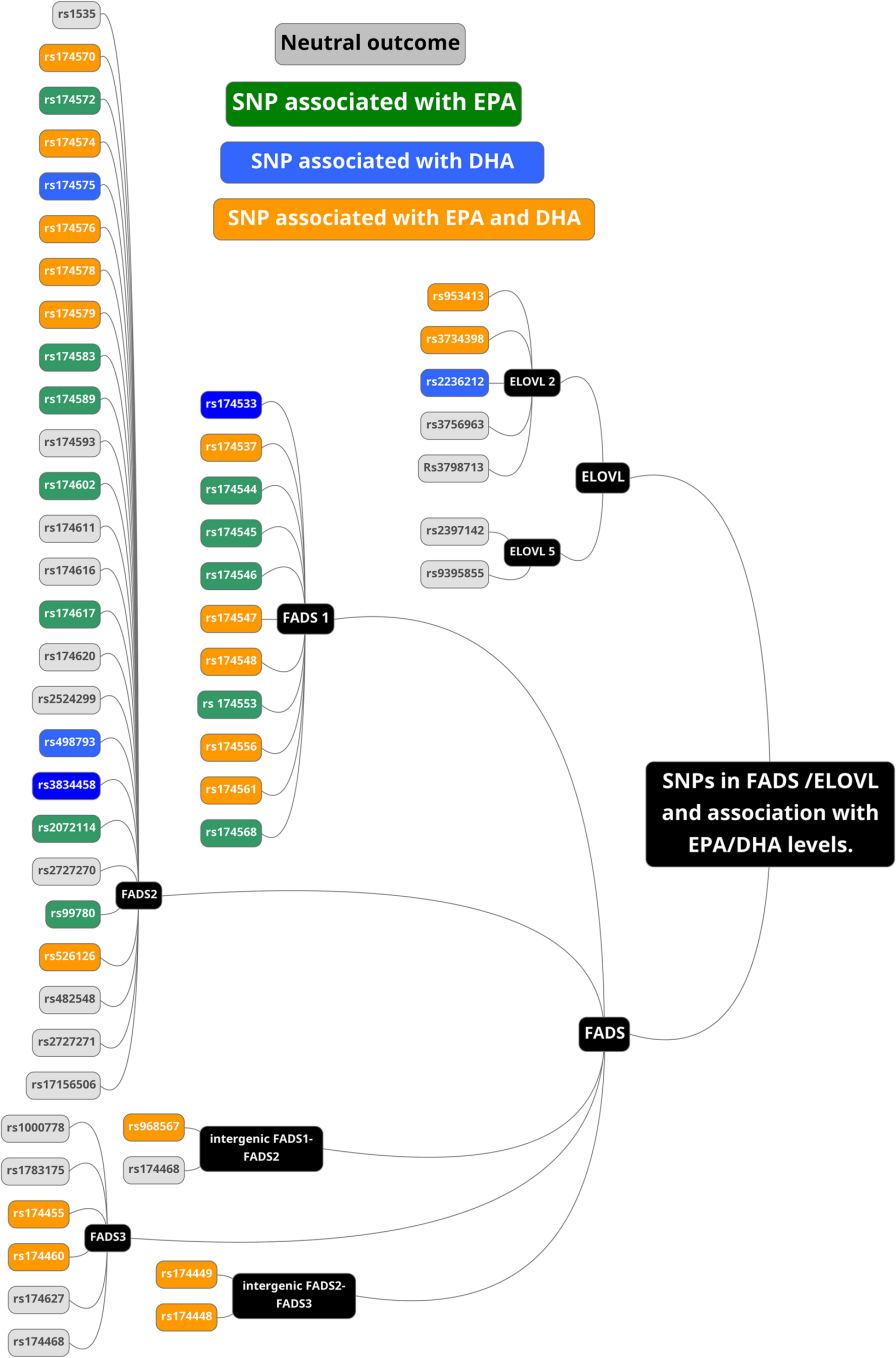
List of studied SNPs in *FADS* / *ELOVL* genes in the selected studies

EPA/DHA were studied in relation to *FADS* polymorphisms in 39 studies (Table 1). Of these, 17 studies reported significant associations between SNPs and EPA levels, and 12 studies with DHA levels. In general, individuals carrying minor (rare) alleles in the *FADS* genes tended to have lower levels of circulating EPA and/or DHA [2, 32–34, 36–50]. However, two studies reported the opposite relationship [51, 52]. These two studies were conducted in China where the minor allele carriers of *FADS1* rs174537 (T) had higher levels of DHA than those carrying the major allele (G) [51, 52]. Altogether, about half of the included studies reported an association between polymorphisms in *FADS* genes and the levels of ω3 LC-PUFA in blood. Figure 3 summarizes all the SNPs found in the selected studies. Green nodes represent SNPs associated with EPA, blue nodes represent SNPs associated with DHA, orange nodes are for the SNPs associated with both EPA and DHA at least in one study and grey is for no reported associations.

**Table 1:** Studies investigating the association between *FADS* /*ELOVL* genetic polymorphisms and EPA/ DHA levels in blood

	**Reference**	**Population size / ethnicity** **(Study site)**	**Measure**	**Gene**	**Studied SNPs**	**Alleles** **(M>m)**	**MAF** **(%)**	**Genetic model**	**EPA/DHA intake**	**Outcome**
**1**	Al-Hilal et al., 2013 [42]	*n*=310 (United Kingdom).	EPA and DHA % in plasma.	*FADS1*	rs174537	G>T	0.3	Dominant	NA	Minor allele carriers had lower EPA and DHA.
					rs174561	T>C	0.27			
				*FADS2*	rs3834458	T>D	0.3			
**2**	Alsaleh et al., 2014 [69]	*n*=310 (United Kingdom)	EPA and DHA % in plasma.	*ELOVL2*	rs3734398	G>T	0.45	Dominant	NA	Minor allele carriers had lower EPA and DHA.
					rs2236212	T>C	0.44			
					rs953413	T>D	0.48			
**3**	Baylin et al., 2007 [50]	*n*=196 for DHA, *n*= 62 for EPA (Costa Rica)	EPA and DHA concentrations in plasma.	*FADS*2	rs3834458	T>D	0.48	Additive	EPA (% of energy) was (0.04 ± 0.04) in cases and (0.04 ± 0.03) in controls, DHA was (0.07 ± 0.06) in both cases and controls	Minor allele carriers had lower EPA and DHA.
**4**	Bernard et al., 2018 [67]	*n*=786 Chinese, Malay and Indian (Singapore).	EPA and DHA concentrations in plasma.	*FADS1*	rs174546	A>G	Minor allele frequency >5%.	Additive	NA	No association.
				*FADS2*	rs2727270	G>A				
					rs174593	A>G				
					rs498793	G>A				
					rs17156506	G>A				
				*FADS3*	rs174450	A>G				
					rs1000778	G>A				
					rs174455	A>G				
**5**	Bokor et al, 2010 [49]	*n*=1144 Caucasians (Europe).	EPA and DHA % in serum.	*FADS1*	rs174546	C>T	0.31	Dominant	NA	Minor allele carriers of *FADS*1-rs174546, *FADS2-*rs174572, and rs174589 had lower EPA levels. No association with DHA.
				*FADS2*	rs968567	C>T	0.16			
					rs174570	C>T	0.11			
					rs174572	C>T	0.23			
					rs2072114	C>T	0.11			
					rs174589	C>G	0.11			
					rs174602	T>C	0.22			
					rs498793	T>C	0.4			
					rs526126	C>G	0.2			
					rs174611	T>C	0.27			
					rs174616	G>A	0.48			
**6**	Carvalho et al., 2019 [65]	*n*=250 Different ethnicities (Brazil).	EPA and DHA % in plasma.	*FADS1*	rs174561	T>C	0.29	Additive	EPA (mg/day) 65.6 ± 73.1 DHA (mg/day) 209.9 ± 211.7	No association.
				*FADS2*	rs174575	C>G	0.22			
					rs3834458	T/D	0.24			
**7**	Coltell et al,. 2020 [2]	*n*= 426, Caucasians (Spain).	Omega-3 concentration in serum (%), DHA % in serum.	*FADS1*	rs174547	T>C	0.29	Additive	NA	Minor allele carriers had lower (EPA+DHA) and DHA concentrations.
**8**	Cribb et al., 2018 [53]	*n*= 187 Europeans (Australia).	EPA % in RBC.	*FADS1*	rs174537	G>T	0.32	Additive	NA	No association.
					rs174547	T>C	0.33			
				*FADS2*	rs174570	C>T	0.12			
					rs174575	C>G	0.28			
					rs498793	C>T	0.33			
					rs3834458	T>D	0.39			
**9**	De la Garza Puentes et al., 2017 [56]	*n*=180 (Spain)	EPA and DHA % in plasma.	*FADS1*	rs174537	G>T	NA	Dominant	EPA (g/d) 0.13 ±0.13 in normal-weight participants and 0.12 ± 0.10 in over-weight /obese participants. DHA (g/d) 0.25 ± 0.20 in normal-weight and 0.27 ± 0.18 in over-weight /obese participants.	No association.
					Rs174545	C>G	NA			
					rs174546	C>T	NA			
					rs174548	C>G	NA			
					rs174553	A>G	NA			
					rs174561	T>C	NA			
					rs174547	T>C	NA			
				*FASD2*	rs1535	A>G	NA			
					rs174575	C>G	NA			
					rs174583	C>T	NA			
					rs99780	C>T	NA			
					rs174602	T>C	NA			
				*ELOVL2*	rs2236212	G>C	NA			
					rs3798713	G>C	NA			
					rs953413	A>G	NA			
				*ELOVL5*	rs2397142	C>G	NA			
					rs9395855	T>G	NA			
**10**	Dumont et al., 2011 [47]	*n*=573 Europeans.	EPA and DHA % in serum PL.	*FADS1*	rs174546	C>T	0.32	Dominant + additive	NA	Minor allele carriers had lower EPA. No association with DHA.
**11**	Fujii et al., 2020	*n*=226 59% Caucasians (Brazil).	EPA and DHA concentrations in plasma.	*FADS1*	rs174546	C>T	NA	Dominant	NA	Minor allele carriers of rs174546 had lower EPA.
				*ELOVL2*	rs953413	A>G	NA			
**12**	Hammouda et al,. 2020 [36]	*n*= 274 (Tunisia).	EPA and DHA concentrations in plasma and RBC.	*FADS1*	rs174556	T>C	0.33	Dominant	NA	No association.
				*ELOVL2*	rs3756963	T>C	0.33			
**13**	Harsløf et al., 2013 [34]	*n*=401 (Denmark).	EPA and DHA % in RBC.	*FADS2*	rs1535	A>G	0.3	Additive	ω3 LC-PUFA (% of energy) 0.8 [0.6, 1.0]	Minor allele carriers of rs174448 had lower EPA. Minor allele carriers of rs174575 had lower levels of DHA.
					rs174575	C>G	0.21			
				*(FADS1-FADS2)*	rs3834458	T>D	0.29			
				*(FADS2-FADS3)*	rs174448	A>G	0.3			
**14**	Hermant et al., 2018 [62]	*n*=540 (France).	EPA and DHA % in RBC.	*FADS1*	rs174547	T>C	0.3-0.32	Additive	NA	No association.
**15**	Hong et al., 2018 [57]	*n*= 122 (Korea).	EPA and DHA % in serum PL.	*FADS1*	rs174537	G>T	0.35	Dominant	NA	No association.
				*FADS2*	rs2727270	C>T	0.3			
**16**	Horiguchi et al., 2016 [35]	*n*=124 (Japan).	EPA and DHA % in plasma and RBC, PL.	*FADS1*	rs174547	T>C	0.39	Additive	EPA (mg/d) TT: 156±112 CT: 153±104 CC: 158±109 DHA (mg/d) TT: 262±149 CT: 260±141 CC: 272±164	No association.
**17**	Huang et al., 2014 [52]	*n*=1158 (China).	EPA and DHA in RBC PL.	*FADS1*	rs174537	G>T	0.45	Dominant, recessive and additive	NA	Minor allele carriers of *FADS1-* rs174537 had higher levels of DHA in patients.
				*FADS2*	rs174575	C>G	0.12			No association with *FADS2.*
				*FADS3*	rs174455	A>G	0.29			Minor allele carriers of *FADS3-*rs174455 had significantly lower levels of EPA in patients.
**18**	Lauritzen et al., 2017 [84]	*n*=765 (Denmark).	DHA % in whole blood.	*FADS2*	rs1535	A>G	0.33	Dominant	NA	No association.
					Rs174448	A>G	0.36			
				*FADS3*	rs174468	G>A	0.42			
				*ELOVL5*	rs2397142	C>G	0.31			
**19**	Li et al., 2013 [51]	*n*= 1015 (China).	EPA and DHA concentrations in plasma.	*FADS1*	rs174537	G>T	0.33	Dominant	NA	Minor allele carriers of rs174537 had lower levels of EPA in patients and higher levels of DHA in controls. Minor allele carriers of rs174460 had lower levels of DHA and higher EPA only in patients.
				*FADS3*	rs174460	T>C	0.48			
**20**	Lu et al., 2012 [41]	*n*= 1246 Caucasians (Netherlands).	EPA and DHA % in plasma CE.	*FADS1*	rs174547	A>G	-	Additive		Minor allele carriers had lower EPA and DHA.
**21**	Malerba et al, 2008 [60]	*n*=658 Caucasians (Italy).	EPA and DHA% in plasma and RBC PL.	*FADS1*	rs174545	G>C	0.29	Additive	NA	No association.
					rs174556	C>T	0.24			
					rs174561	T>C	0.25			
				*FASD2*	rs3834458	T>D	0.3			
					rs174570	C>T	0.11			
					rs2524299	A>T	0.09			
					rs174583	C>T	0.32			
					rs174589	C>G	0.2			
					rs498793	G>A	0.41			
					rs174611	T>C	0.25			
				*FADS3*	rs1783175	T>C	0.1			
					rs174627	C>T	0.13			
**22**	Metelcová et al., 2021 [48]	*n*=670 Caucasians (Czech Republic).	EPA % in plasma PL and TG.	*FADS1*	rs174546	C>T	0.32	Additive	NA	There was just a significant difference in boys’ level of EPA.
					rs174537	G>T	0.32			
**23**	Murff et al., 2022 [58]	*n*= 126 61% Caucasians (USA).	EPA and DHA % in RBC.	*FADS1*	rs174537	G>T	NA	Additive	NA	No association.
**24**	Muzsik et al., 2018 [61]	*n*= 125 (Poland).	EPA and DHA concentrations in RBC.	*FADS1*	rs174556	C>T	NA	Dominant	EPA (mg/d) 59.3±10.4 DHA (mg/d) 139.4± 21.6	No association.
					Rs174547	T>C	NA			
					rs174561	T>C	NA			
				*FADS2*	rs3834458	T>D	NA			
**25**	Nita et al., 2020 [59]	*n*= 416 (Japan).	EPA and DHA % in RBC.	*FADS1*	rs174547	T>C	0.39	Additive	NA	Minor allele carriers had lower DHA. No association with EPA.
**26**	Khamlaoui et al., 2020 [38]	*n*=295 (Tunisia).	EPA and DHA concentrations in plasma.	*FADS1*	rs174556	T>C	NA	Additive	NA	Minor allele carriers of *FADS1* had lower DHA. Minor allele carriers of *FADS2* and *ELOVL2* had lower EPA.
				*FADS2*	rs174617	T>C	NA			
				*ELOVL2*	rs3756963	T>C	NA			
**27**	Kim et al., 2011 [54]	*n*=567 (Korea).	EPA and DHA % of serum PL.	*FADS1*	rs174537	G>T	0.32	Dominant	NA	No association.
				*FADS2*	rs174575	G>T	0.082			
					rs2727270	C>T	0.24			
				*FADS3*	rs1000778	C>T	0.29			
**28**	Koletzko et al., 2011 [33]	*n*= 4457 Caucasians (United Kingdom).	EPA and DHA % in RBC.	*FADS1*	rs174548	C	0.31	Additive	NA	Minor allele carriers of all *FADS* polymorphisms had lower EPA and DHA. Only rs174570, rs2727271, rs174602, rs498793 were not associated with EPA and rs2727271, rs174602 were not associated with DHA.
					Rs174556	A	0.3			
					rs174561	G	0.3			
				*FADS1-FADS2*	rs3834458	D	0.33			
					rs968567	T	0.17			
				*FADS2*	rs174570	T	0.13			
					rs174574	A	0.34			
					rs2727271	T	0.12			
					rs174576	A	0.34			
					rs174578	A	0.34			
					rs174579	T	0.22			
					rs174602	G	0.2			
					rs498793	T	0.39			
					rs526126	G	0.18			
				*FADS2-FADS3*	rs174448	C	0.36			
					rs174449	C	0.35			
				*FADS3*	rs174455	C	0.35			
**29**	Roke and Mutch 2014 [32]	*n*= 12 Caucasians (Canada).	EPA and DHA concentrations in serum and RBC.	*FADS1*	rs174537	G>T	-	Dominant	NA	Minor allele carriers of rs174537 had lower EPA in serum but not in RBC. No association with DHA.
				*FADS2*	rs174576	C>A	-			
**30**	Roke et al., 2016 [46]	*n*= 26 Caucasians (Canada).	EPA and DHA % in RBC.	*FADS2*	rs174576	C>A	-	Dominant	NA	Minor allele carriers had lower EPA. No association with DHA.
**31**	Schaeffer et al., 2006 [43]	*n*=727 Caucasians (Germany).	EPA and DHA % in serum PL.	*FADS1*	rs174544	C>A	0.29	Additive	NA	Minor allele carriers of rs174544, rs174553, rs174556, rs174561, rs174568, rs968567, rs99780, rs174570, rs2072114, rs174583 and rs174589 had lower EPA. No association with DHA.
					rs174545	C>G	0.33			
					rs174546	C>T	0.34			
					rs174553	A>G	0.33			
					rs174556	C>T	0.27			
					rs174561	T>C	0.28			
				*FADS1-FADS2*	rs174568	C>T	0.32			
					rs3834458	T>D	0.32			
					rs968567	G>A	0.16			
				*FADS2*	rs99780	C>T	0.34			
					rs174570	C>T	0.13			
					rs2072114	A>G	0.15			
					rs174583	C>T	0.34			
					rs174589	G>C	0.17			
					rs174602	A>G	0.18			
					rs526126	G>C	0.18			
					rs174620	T>C	0.43			
					rs482548	C>T	0.09			
**32**	Scholtz et al., 2015 [44]	*n*=205 (Hispanic /non-Hispanic) (USA).	DHA concentration in RBC PL.	*FADS1*	rs174553	A>G	0.28	Additive	NA	Minor allele carriers of rs174533 had lower DHA. No association with rs174575.
				*FADS2*	rs174575	C>G	0.27			
**33**	Schuchardt et al., 2016 [40]	*n*= 111 Caucasians (Germany).	EPA and DHA % in RBC.	*FADS1*	rs174548	C>G	0.21	Additive	NA	Minor allele carriers of rs174548 had lower DHA. No association with rs1535.
				*FADS2*	rs1535	A>G	0.24			
**34**	Tanaka et al., 2009 [37]	InCHIANTI study *n*=1075 Caucasians (Italy) GOLDN study *n*=1076 Caucasians (US).	EPA and DHA % in plasma.	*FADS1*	rs174537	G>T	NA	Additive	NA	Minor allele carriers of rs174537 had lower EPA. Minor allele of rs953413 associated with lower DHA. Minor allele of rs953413 associated with higher in InCHIANTI study only.
				*ELOVL2*	rs953413	G>A	NA			
**35**	Tandon et al., 2021 [85]	*n*=112 (Mexico).	EPA and DHA concentrations in plasma.	*FADS 2*	rs174602	C>T	NA	Dominant	EPA (g/d) Carriers: 0.02 [0.01–0.05] Non-carriers: 0.01 [0.01–0.03] DHA(g/d) Carriers: 0.06 [0.04–0.13] Non-carriers: 0.05 [0.03–0.08]	No association.
**36**	Xie and Innis 2008 [64]	*n*= 67 Caucasians (Canada).	EPA and DHA in plasma and RBC PL.	*FADS1*	rs174553	A>G	NA	Additive	EPA (mg/d) 76.1 ± 65.6 DHA(mg/d) 118 ± 122	No association.
					Rs99780	C>T	NA			
				*FADS2*	rs174575	C>G	NA			
					rs174583	C>T	NA			
**37**	Yao et al., 2015 [45]	*n*=752 (China).	EPA and DHA % in serum.	*FADS1*	rs174545	C>G	0.3	Additive	NA	Minor allele carriers of rs174602, rs174545 and rs2072114 had lower EPA. No association with DHA.
				*FADS2*	rs2072114	A>G	0.21			
					rs174602	A>G	0.26			
					rs174616	C>T	0.16			
**38**	Yeates et al., 2015 [66]	*N*=1622 Different origins (Seychelles).	EPA and DHA concentrations in serum.	(*FADS1-FADS2*)	rs3834458	T>D	NA	Additive	NA	No association.
				*FADS2*	rs174575	C>G	NA			
**39**	Zec et al., 2020 [86]	*n*= 88 Caucasians (Serbia).	EPA and DHA levels in plasma PL.	*FADS2*	rs174593	T > C	NA	Dominant	NA	No association.
					Rs174616	G > A	NA			
					rs174576	C > A	NA			
**40**	Zietmann et al, 2010 [87]	*n*=2066 Caucasians (Europe)	EPA and DHA % in RBC.	*FADS1*	rs174546	C>T	>0·05	Additive	EPA (% of energy) 0.12 DHA (% of energy) 0.21.	No association.

Allele frequencies are as reported in the studies Genetic model: dominant (MM compared to Mm + mm), additive (MM compared to mm and to Mm), recessive (MM + Mm compared to mm)

*MAF* Minor allele frequency, *D* Deletion, *n* Number of subjects in the study, *FADS* Fatty Acid Desaturase, *ELOVL* Elongation of Very Long chain fatty acids, *PL* Phospholipids, *RBC* Red blood cells, *EPA* Eicosapentaenoic acid, *DHA* Docosahexaenoic acid, *TG* Triglycerides, *CE* Cholesterol esters, *NA* Not available

#### FADS1 gene

Among the 39 studies, 11 SNPs in the *FADS1* gene were reported: rs174533, rs174537, rs174544, rs174545, rs174546, rs174547, rs174548, rs174553, rs174556, rs174561, and rs174568 (Fig. 3). For rs174533, the authors reported DHA levels only [44]. All other SNPs were associated with EPA levels, although this result was not consistently found in all studies. In contrast, only 6 SNPs were associated with DHA levels. Among the different studied SNPs, *FADS1*-rs174544 and rs174568 were investigated in one paper only [43] and will therefore not be discussed further since the results were not independently validated.

**Fig. 4:**
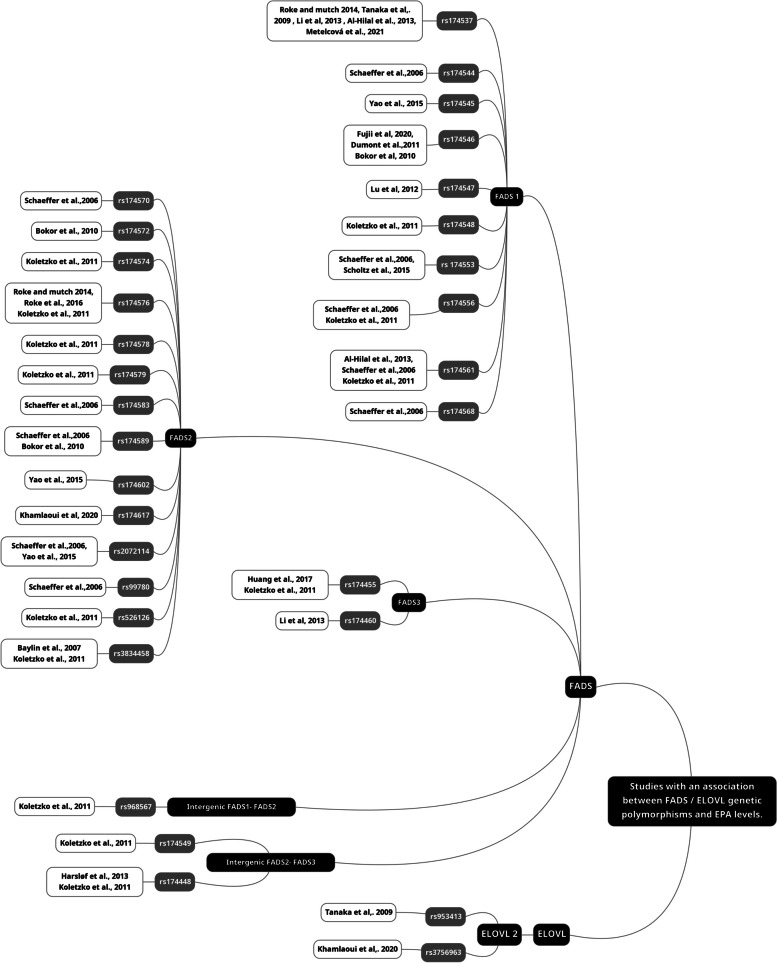
List of studies with an association between *FADS* / *ELOVL* genetic polymorphisms and EPA levels

**Fig. 5:**
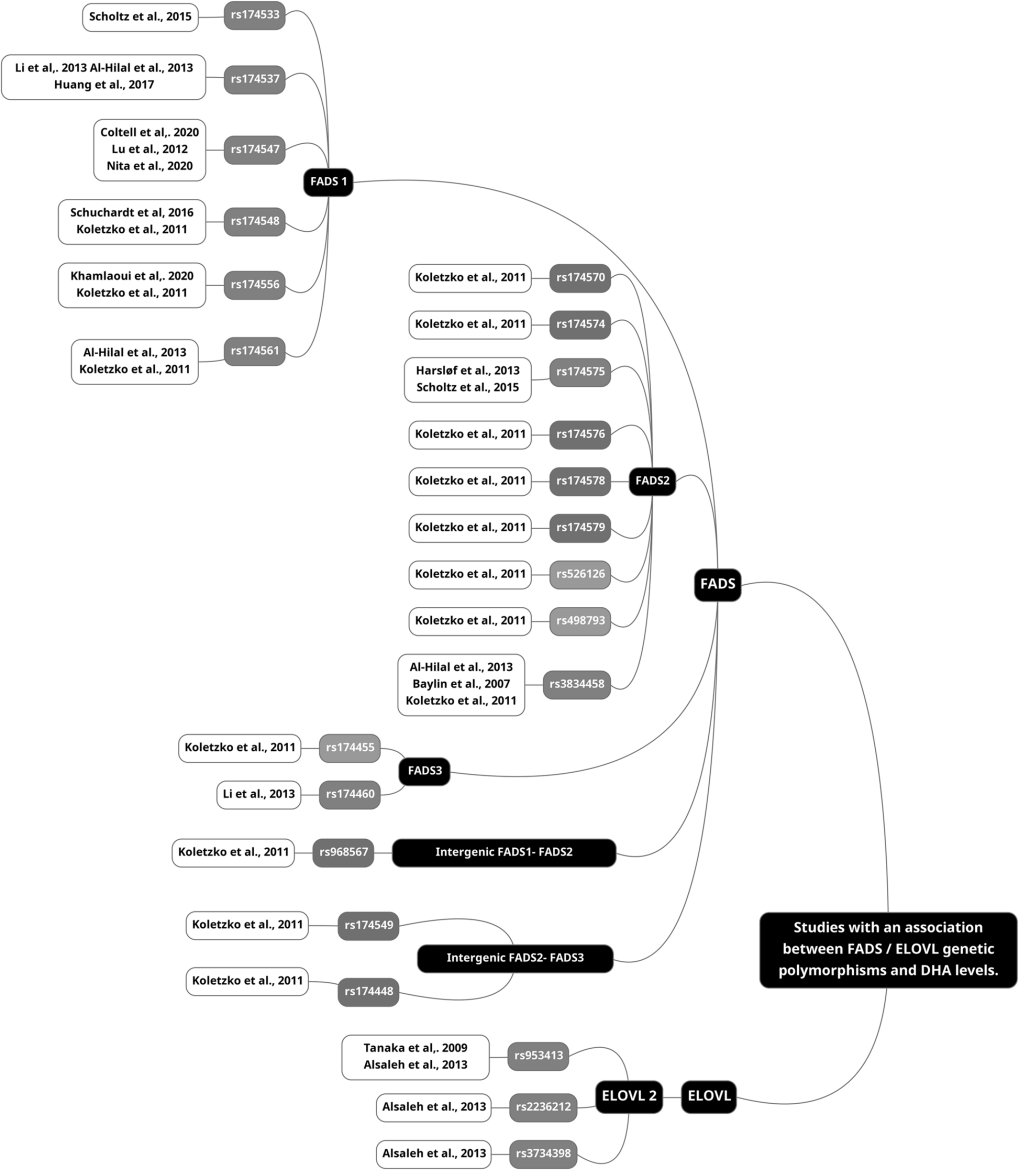
List of studies with an association between *FADS* / *ELOVL* genetic polymorphisms and DHA levels

**Fig. 6:**
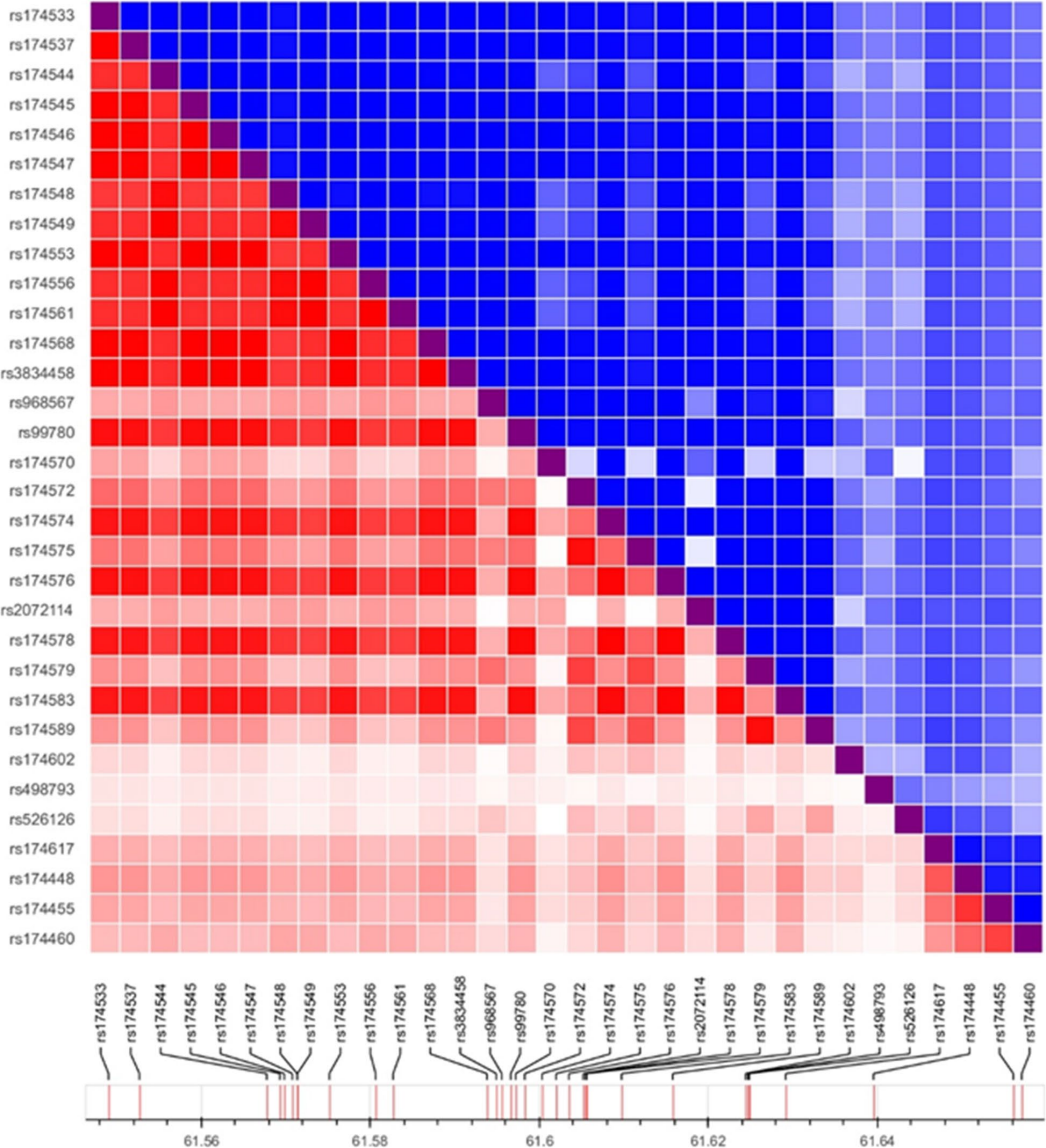
Pairwise linkage disequilibrium D’ and R^2^ plots of the *FADS* SNPs associated with EPA/DHA in selected studies

Figure 4 summarizes the studies reporting significant associations between SNPs and EPA levels, and Fig. 5 summarizes those with DHA levels. The majority of *FADS1* SNPs were in strong linkage disequilibrium (LD), except for rs174548, which displayed LD coefficients < 0.8 with rs174533, rs174537, rs174544, rs174545, rs174546, rs174547, rs174553, rs174556, rs174561, and rs174568 (Fig. 6) as established with LDlink.

Regarding the other SNPs in the *FADS1* gene, 11 studies investigated the rs174537 polymorphism. In all these studies, (G) was the major allele and (T) the minor allele. The minor allele frequencies differed by populations and varied between 0.3 in Europeans to 0.45 in east Asians [52]. *FADS1*- rs174537 was in high LD (R^2^ > 0.8) with 10 SNPs in the *FADS1* gene and 6 SNPs in the *FADS2* gene (Fig. 6). Five studies showed that individuals carrying the minor (T) allele in rs174537 had lower EPA levels in plasma [37, 42, 48, 51] and serum [32] compared to those carrying the major G allele. However, seven studies did not report such an association [53–58]. Blood DHA was also associated with rs174537 in three independent studies [42, 51, 52]. In a healthy cohort used by Hilal et *al.,* 2013 [42], minor allele carriers had lower levels of DHA in plasma, while in two other independent papers in East Asians with coronary artery disease and Type 2 diabetes [51, 52], those carrying the minor allele had higher levels of DHA than those with the major allele. When EPA and DHA were measured in RBCs, the results were less consistent than when analyses were performed in plasma or serum [32, 53].

Among the other *FADS1* SNPs, 7 studies investigated rs174556 [33, 36, 38, 43, 59–61] (Table 1). Only two studies found that individuals carrying the minor (A) allele had lower serum EPA levels than those with the major (T) allele [33, 43]. None of the other studies reported a significant difference between minor and major allele carriers. It is notable that there was a difference in major and minor allele frequencies for this SNP between populations from different countries. For instance, in the Tunisian population, (T) is the major allele [36, 38], whereas in individuals of European descent, (C) is the major allele [43, 60, 61]. Another study of a British cohort listed (A) as a minor allele but did not report the major allele of any of the studied SNPs [33]. In this specific study, the minor allele was associated with lower levels of both EPA and DHA. This shift in the frequency of T and C alleles in rs174556 between ethnicities thus warrants caution when directly comparing studies to draw a clear conclusion about the presence or the absence of relationship with blood EPA and DHA.

Eight studies investigated the rs174547 [2, 35, 41, 53, 56, 59, 61, 62], where (T) was the major allele and (C) the minor allele. Three of the studies investigating this SNP reported that carriers of the major (T) allele had higher DHA levels [2, 41, 59], and two studies reported lower EPA levels in minor allele carriers [2, 41]. However, five studies did not report any significant differences in ω3 LC-PUFA in those carrying the minor or the major allele [35, 53, 56, 61, 62].

*FADS1*-rs174561 is another SNP in the *FADS* gene cluster that was investigated in 8 studies. These past studies reported that (T) was the major allele and (C) was the minor allele. Three studies reported lower EPA levels in those carrying the minor (C) allele [33, 42, 63] and two of them reported lower DHA levels [33, 42].

#### FADS2 gene

Twenty-six SNPs within the *FADS2* gene were investigated across 27 studies (Table 1, Fig. 3). Overall, individuals carrying the minor alleles in any of the following 15 SNPs had lower levels of circulating EPA (Fig. 4) or DHA (Fig. 5): rs174570, rs174572, rs174574, rs174575, rs174576, rs174578, rs174579, rs174583, rs174589, rs174602, rs174617, rs2072114, rs99780, rs3834458 and rs498793. 7 SNPs were associated with both EPA and DHA, whereas 5 were associated with EPA only (Fig. 4) and 2 SNPs (rs174575 and rs498793) with DHA only (Fig. 5) [33, 34, 44]. The most widely studied SNPs within the *FADS2* gene are rs174575 and rs3834458. These two SNPs are not in high LD (*r*^2^=0.552). There were more studies reporting lower DHA levels for minor allele carriers than EPA. Nine studies investigated the rs174575 SNP [34, 44, 52–54, 56, 64–66], with only two reporting a significant association with DHA [34, 44]. None of the selected studies reported associations between rs174575 and EPA levels.

*FADS2* rs498793 was investigated in 5 studies [33, 49, 53, 60, 67] and only Koletzko *et al.* [33] reported that minor allele carriers (T) had lower levels of DHA. None of the studies found an association with EPA. Finally, rs174574, rs174578, and rs174579 in *FADS2* were investigated by a single group [33]. EPA and DHA were lower in minor allele carriers for all these SNPs. rs174572 was investigated by another independent group [49], who did find lower EPA levels in minor allele carriers (T) .

#### FADS3 gene

Six SNPs in the *FADS3* gene were examined across seven studies (Table 1): rs1000778, rs1783175, rs174455 and rs174460, rs174627, rs174468 (Fig. 3). Among these SNPs, only rs174455 and rs174460 were found to be associated with EPA and DHA [33, 52]. These two SNPs were in moderate LD (*r*^2^ = 0.746). Minor allele carriers (G) of rs174455 had lower levels of EPA and DHA [33], while in another study [47], they only showed lower levels of EPA. *FADS3*-rs174460 was only investigated in one paper [51], where minor allele carriers (C) had lower DHA levels but higher EPA levels.

### *ELOVL* genetic polymorphisms and EPA/DHA status in blood pools

**Fig. 7:**
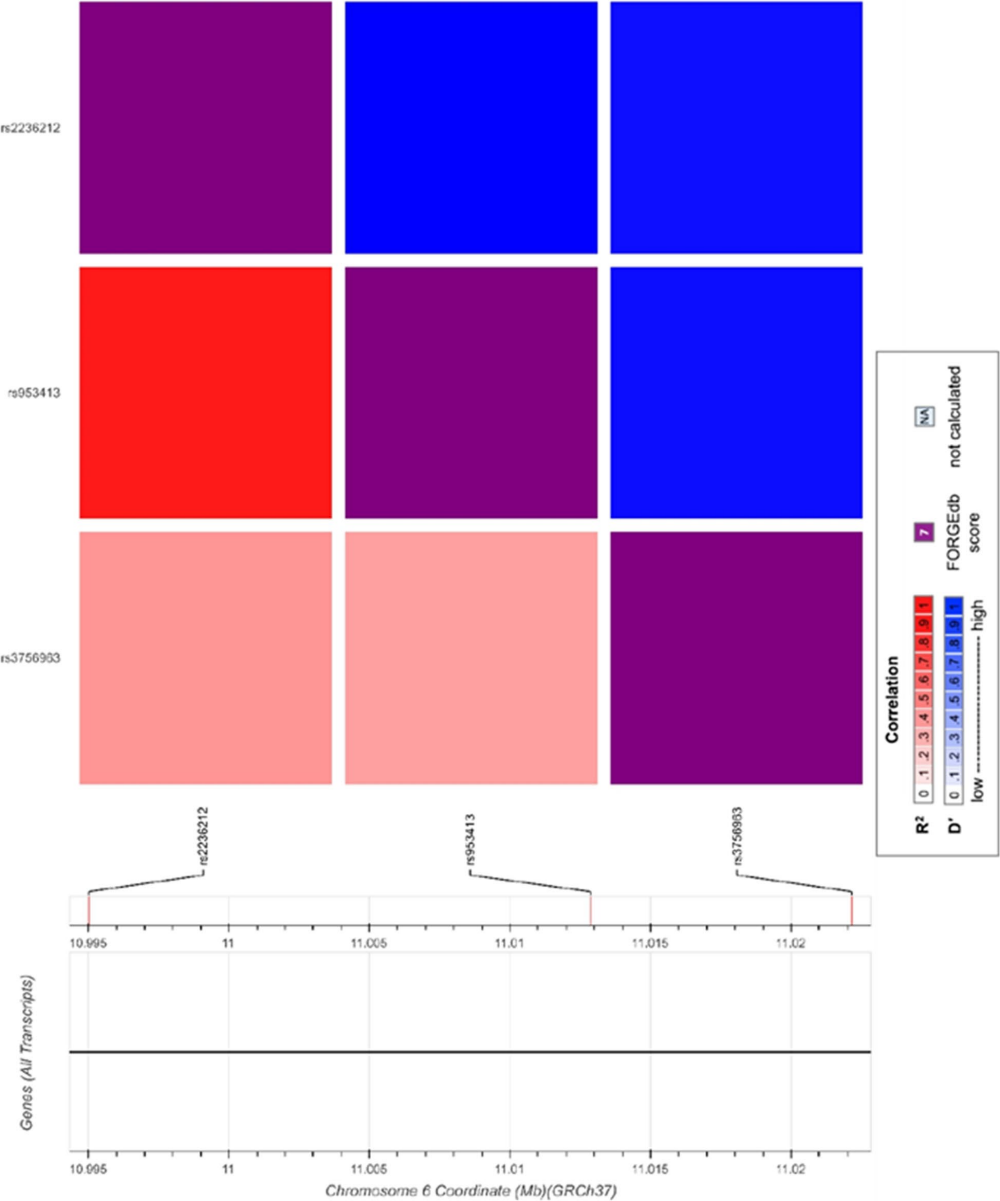
Pairwise linkage disequilibrium D’ and R^2^ plots of the *ELOVL* SNPs associated with EPA/DHA in selected studies

Seven different *ELOVL* polymorphisms were investigated in 7 studies (Table 1). *ELOVL2*-rs2236212 was in high LD with *ELOVL2-*rs953413, (*R*^2^= 0.9, Fig. 7). Overall, five different SNPs in *ELOVL2* were investigated: rs953413, rs3734398, rs2236212, rs3756963, and rs3798713. As for *ELOVL5,* two studies examined the genetic variants rs2397142 and rs9395855 and their association with blood levels of EPA and DHA. However, neither study reported a difference in blood levels of EPA and DHA between individuals carrying the major and minor alleles of these SNPs [56, 68].

For *ELOVL2,* two SNPs were associated with EPA (Fig. 4) and three with DHA (Fig. 5). For rs953413, the individuals carrying the minor (A) allele had significantly lower DHA in both Italian and US cohorts [37]. This was replicated in a UK population [69]. Minor allele carriers of *ELOVL2* rs953413 (A) had higher EPA levels and lower DHA levels [37]. Other SNPs (rs3734398 and rs2236212) were investigated and those with the minor allele had lower DHA levels [69]*.* Finally, in two studies conducted in Tunisia, results on the rs3756963 were divergent, where minor allele carriers (C) had lower EPA levels in the study by Khamlaoui et *al.,* [38] while there was no association found in the study by Hammouda et *al*., [36]. The study populations varied, with the former study including included obese and normo-weighted individuals, and the latter study including controls and patients with Alzheimer's disease. This difference in study populations may have played a role in the disparate findings observed.

## Discussion

In this scoping review we investigated whether EPA and DHA blood levels were modified by polymorphisms in the *FADS* and *ELOVL* genes. The objective was to assess whether these genetic variants could account for the observed variations in EPA and DHA levels in the bloodstream.

Hence, the idea was to identify one or two SNPs, and not haplotypes, as they allow for a more focused analysis of specific genetic differences, while haplotypes provide information about the combination of multiple SNPs.

Our results support that more SNPs are associated with EPA blood levels than with DHA. It is notable that many of the SNPs in the *FADS* gene are in high LD. Furthermore, 3.6 % of the SNPs in *FADS* genes are missense or nonsense variants (i.e., *FADS1*- rs174544, rs174545, and rs174546 are missense SNPs) and can thus change the amino acid sequence of desaturases. These SNPs have the potential to affect protein translation by altering the binding of regulatory proteins or microRNAs that control mRNA stability and translation efficiency [70], or they can change the enzymatic activity of the desaturases [43]. This may support, in part, their association with EPA/DHA levels. *FADS1*- rs174544, rs174545, and rs174546 are also in strong LD with all *FADS1* SNPs, except for *FADS1*-rs174548, where they were in moderate LD with an R^2^ value between 0.7 - 0.8. These findings are consistent with the conclusions drawn in the review paper, where minor allele carriers, for example of *FADS1-*rs174544 (A), had lower levels of desaturase products, mainly EPA (Fig. 4).

For the selected studies, some of the SNPs in *FADS*/*ELOVL* were significantly associated with higher level of substrates such as linoleic acid, eicosadienoic acid, dihomo-γ linolenic acid and ALA as reported in Schaeffer et al., [63] and lower level of products such as γ-linolenic acid, arachidonic acid, adrenic acid, EPA, docosapentaenoic acid and DHA. Most of these SNPs were in high LD. Therefore, it’s not clear whether one SNP has a dominant role over the others. Indeed, some of these genetic variants are in gene regions that may support their dominant role. For example, this is the case with the *FADS1*-rs174561 SNP that is located in a cytosine-phosphate-guanine (CpG) island where there is a transcription factor-binding site in the *FADS1* gene [63]. It is worth noting that the minor allele in rs174561 is a (C) nucleotide, which may affect the functionality of the CPG island [63]. This is important, since CpG are important sites of methylation in mammals that can influence gene expression [72]. Therefore, a methylated (C) nucleotide in minor allele carriers could result in a transcriptional repression since this epigenetic reaction directly inhibits the binding of certain transcription factors, as confirmed by Hoile et *al.,* 2014 [73]. This group also showed that supplementation with ω3 LC-PUFA changed the methylation status of the CpG island in *FADS* and *ELOVL* genes.

Another SNP that was highly studied and associated with ω3 LC-PUFA levels is the *FADS1-*rs174537 variant. This SNP is also located within a gene region that has been identified as a strong genetic proxy for DNA methylation. Specifically, it is situated in a putative promoter region of the *FADS* gene cluster. Notably, the methylation status of this locus has the potential to directly impact gene expression [74]. DNA methylation status varied by 40% between homozygotes at rs174537 (44% for GG genotype versus 84% TT genotype), where (T) is the minor allele and (G) is the major allele in this SNP. According to *in vitro* studies with human hepatocytes, *FADS1* metabolism is also allele-specific, depending on the genotype of rs174537. Specifically, the expression of *FADS1* and *FADS2* genes were significantly reduced in hepatocytes derived from individuals homozygous for the minor allele at *FADS1-*rs174537 (i.e., TT) [75, 76].

The measure of fatty acids in the blood is an indirect measure of the elongation and desaturation efficiency of the *FADS* and *ELOVL* genes. A measure of the actual desaturase activities by measuring enzyme activity would have been more accurate to support the data [57]. Indeed, these blood levels might be influenced by other factors such as ethnicity, sex, diet and physio pathological factors. In addition to investigating blood fatty acids that are more variable than that of other tissues, some teams have investigated the same question in adipose tissues. For example, one study examined associations between *FADS* SNPs and fatty acid composition of subcutaneous adipose tissue (SAT) [76]. The results have consistently shown that the *FADS1*- rs174537 genotype is associated with distinct SAT fatty acid profiles. Specifically, individuals carrying the major (G) allele had higher levels of arachidonic acid and lower levels of dihomo-γ-linolenic acid [76]. Similar findings were found by another group that had also demonstrated that minor allele carriers of *FADS1-* rs174556 (C>T), *FADS1-* rs2524299(A>T)*, FADS1/FASD2* intergenic rs3834458 (T>deletion) and *FADS2-*rs174570 (C>T) had lower levels of EPA in SAT [77]. The advantages of using the adipose tissue as a proxy measure of the conversion rate is that it better captures long-term dietary intakes, since SAT fatty acid turnover is slow, there is an absence of recall bias, and response to conditions of acute disease [76]. Hence, it could be speculated that this tissue is more appropriate to investigate the relationship between *FADS* and *ELOVL* polymorphisms and enzymatic activity. However, it is important to outline that EPA and DHA are stored at very low concentrations in the adipose tissue [18]. The role of the adipose tissue is to serve as a reservoir of fat for the body and it therefore does not reflect the dynamic exchange between organs and tissues like the blood.

In this review, we also noticed that allele frequency differed among the populations, likely due to different ethnic backgrounds. For instance, for *FADS1* rs174547,(C) was the major allele and (T) the minor allele in 6 studies [35, 53, 56, 59, 61, 62], while in another country (A) was the major and (G) the minor allele [41]. This difference in minor alleles frequencies between populations made it difficult to state that minor allele frequency of this SNP is associated with higher or lower level of EPA and DHA considering the genetic variation between studies. Another factor influencing the levels of blood EPA and DHA is age. Some of the present authors previously showed that with aging, levels of ω3 LC-PUFA, more specifically EPA plasma levels, are higher in older compared to younger individuals [71, 78, 79]. Coltell et *al.,* [2] found that the minor (C) allele of rs174547 was associated with lower serum ω3 LC-PUFA concentrations and they suggested that the age of the participants (from 55 to 75 years old ) could explain why their result differed from that of other groups investigating the same SNP and reporting no association with the EPA or DHA blood levels. We noticed that apart from one study conducted in an older Japanese population (80 y) with high fish intake, the other studies investigated this SNP in participants below the age of 70 y [53, 56, 61, 62]. Hence, the point made here by Coltell *et al*., suggests that during aging, where levels of EPA are usually higher in older participants compared to younger, carrying the minor allele of rs174547 (C) could reduce the levels of EPA, which in the long term, could increase the risk of cardiovascular disease [9, 10] and cognitive decline [7, 8] as these diseases’ risks are associated with lower EPA or DHA blood levels.

Genetic variations in the *FADS* and *ELOVL* genes are factors that can influence the circulating levels of EPA and DHA. However, other factors also play a role in determining the levels of these fatty acids. One important factor is the dietary intake of EPA and DHA, as these fatty acids are primarily obtained through dietary consumption [23]. Additionally, gender has also been identified as a factor modulating the levels of DHA since there are differences in desaturases and elongases enzymes activity between males and females [80]. Kitson et al., 2012 [81] and Extier et al., 2010 [82] both demonstrated higher DHA concentrations in female rats compared to males. Kitson et al.,2012 [81] specifically identified higher expression of desaturase and elongase enzymes in female rats. As compared with male rats, the livers of female rats contained 1.0, 1.4, and 1.1fold higher D5D, D6D and elongase 2 mRNA, respectively. Overall, it might be difficult to dissect the exact contribution of each factors to the plasma level of EPA and DHA hence requiring a better comprehensive understanding of genetic, dietary, and physiological factors to fully understand and predict circulating levels of EPA and DHA.

This scoping review has strengths and limitations. Compared to other reviews on *FADS* and *ELOVL* polymorphisms, this review focused specifically on reporting genetic variants associated with ω3 LC-PUFA levels. Another strength of this review paper is that it encompasses a wide range of countries that have conducted research on *FADS*/*ELOVL* polymorphisms, providing ample material to draw comprehensive conclusions. Additionally, conducting such reviews is important since it can help identify gene polymorphisms associated with lower circulating ω3 LC-PUFA. Clinical studies can benefit from such findings to directly target the SNPs that are determinant in ω3 LC-PUFA metabolism, and to examine whether there are any interactions between these SNPs, since genes operate more as highly connected networks than independently. This review also has limitations. We decided not to use a checklist (study quality, study bias, confounding and selection bias, power of the study, etc.) to determine the quality of the papers to include in this review for several reasons. First, the number of studies remains quite limited and using a checklist would have removed studies we considered to be important. Second, we noticed that these tools could rank a paper very high in quality despite having serious flaws in their design. Using our judgement in a previous review, we excluded some papers that, with a checklist tool, were ranked very high in quality but had potential fraudulent data [83]. Another limitation of this review is the number of different SNPs in the *FADS* genes. Indeed, for a same SNP, not all studies had the same minor and major allele. This has complicated our interpretation because we couldn’t pool together all the studies with the same SNP. Moreover, since there is no consensus on the most important SNPs in the *FADS* genes, each SNP had a limited number of studies, and we included studies with different designs and populations background to be tested.

We also acknowledge that the number of studies investigating the *ELOVL* polymorphisms is very limited, which impacted our ability to draw conclusions regarding associations between variants in *ELOVL* genes with EPA and DHA levels.

## Conclusions

In this review, we highlighted that there are many SNPs in the *FADS* genes that are strongly and consistently associated with ω3 LC-PUFA levels. Despite large variability in results, evidence in the literature supports that there are specific SNPs in *FADS* genes that have strong associations with circulating levels of EPA and DHA. We did find that those carrying the minor allele in the *FADS1* rs174537 SNP seem to have consistently lower blood/plasma/serum EPA and DHA levels. This association was generally more consistent for EPA than DHA. Hence, understanding the genetic background is important to target specific EPA/DHA nutritional or supplementation recommendations for individuals, and will therefore have an important role in precision nutrition approaches related to ω3 LC-PUFAs.

## Data Availability

A detailed description of the data used to support these findings can be found within the article.

## Abbreviations

A: Adenosine
ALA: Alpha linolenic acid
CE: Cholesterol ester
C: Cytosine
CpG: Cytosine phosphate guanine
D: Deletion
DHA: Docosahexaenoic acid
DNA: Deoxyribonucleic acid
D6D: Delta 6 desaturase
D5D: Delta 5 desaturase
DPA: Docosapentaenoic acid
EPA: Eicosapentaenoic acid
ETA: Eicosatetraenoic acid
*ELOVL*: Elongation of very long chain fatty acids
G: Guanine
LC-PUFA: Long chain polyunsaturated fatty acids
LD: Linkage disequilibrium
MAF: Minor allele frequency
n: Number of subjects in the study
NCBI: National center of biotechnology information
PL: Phospholipids
RBC: Red blood cells
SNPs: Single nucleotide polymorphism
STA: Stearidonic acid
T2D: Type 2 Diabetes
T: Thymine
TG: Triglycerides
